# Interchange between grooming and infant handling in female Tibetan macaques (*Macaca thibetana*)

**DOI:** 10.24272/j.issn.2095-8137.2018.049

**Published:** 2018-06-28

**Authors:** Qi Jiang, Dong-Po Xia, Xi Wang, Dao Zhang, Bing-Hua Sun, Jin-Hua Li

**Affiliations:** 1 School of Resource and Environmental Engineering, Anhui University, Hefei Anhui 230601, China; 2 School of Life Science, Anhui University, Hefei Anhui 230601, China; 3 School of Life Science, Hefei Normal University, Hefei Anhui 241000, China

**Keywords:** Tibetan macaques (*Macaca thibetana*), Interchange, Infant handling, Grooming, Biological market theory

## Abstract

In some non-human primates, infants function as a social tool that can bridge relationships among group members. Infants are a desired commodity for group members, and mothers control access to them. The biological market theory suggests that grooming is widespread and represents a commodity that can be exchanged for infant handling. As a limited resource, however, the extent to which infants are interchanged between mothers (females with an infant) and non-mothers (potential handlers, females without an infant) remains unclear. In this study, we collected behavioral data to investigate the relationship between grooming and infant handling in free-ranging Tibetan macaques (*Macaca thibetana*) at Mt. Huangshan, China. Our results showed that females with infants received more grooming than females without infants. After her infant was handled, mother females received more grooming than they did during daily grooming interactions. However, with the increasing number of infants within the social group, both the grooming that mothers received and the grooming that non-mothers invested for handling infants decreased. We also found that non-mothers invested more time in grooming to gain access to younger infants than older infants. Our results provide evidence that infants are social commodities for both mother and non-mother females. Mothers use infants for obtain grooming and non-mothers use grooming to gain access to infants. The current study implies a bidirectional and complex interchange pattern between grooming and infant handling to compensate for the dyadic grooming disparity in non-human primates.

## INTRODUCTION

While female attraction to infants represents a common feature in non-human primate species, maternal responses to infant handling show a certain degree of variability (Maestripieri, 1994; Nicolson, 1987). In some species, such as Asian colobines, females allow their newborn infants to be held frequently and carried for long durations by other group members (infant caretaking) (Nicolson, 1987; Stanford, 1992). In other species, such as baboons and macaques, mothers are much more restrictive in allowing access their young offspring (Altmann, 2002; Nicolson, 1987), despite persistent attempts by group members to interact with their infant (Frank & Silk, 2009; Gumert, 2007a; Henzi & Barrett, 2002). It is well documented that infants can be used as social tools to buffer agonistic interactions (Dunbar, 1984; Taub, 1980) and to facilitate social bonds (Dunayer & Berman, 2017; Hsu et al., 2016; Manson, 1999). Therefore, the infant handling concept may be indicative of the behavioral pattern that exists between an infant and a non-mother group member (Hsu et al., 2016; Jin et al., 2015; Maestripieri, 1994).

In recent decades, studies on the social function of infant handling within the framework of the biological market theory have gained increasing interest (Ueno, 2017; Wei et al., 2013; Yu et al., 2013). Biological market theory proposes that the infant can be considered as a social commodity, and thus interchange relationships between infant handling and behavioral commodities may exist. For example, in chacma baboons (*Papio cynocephalus ursinus*), Henzi & Barrett (2002) proposed that female A will groom female B more often if female B has a newborn infant in order to gain access. Yu et al. (2013) described similar findings in golden snub-nosed monkeys (*Rhinopithecus roxellana*), indicating that infant handling might be interchanged for grooming. Infant handling is also reported as a social commodity to interchange for affiliation, e.g., embracing in spider monkeys (*Ateles geoffroyi yucatanensis*) (Slater et al., 2007).

Grooming is a measure of affiliative social relationships among non-human primates (Dunbar, 2010). In some species, grooming accounts for as much as 10%–20% of an individual’s total daily activity budget (Lehmann et al., 2007). Previous studies have demonstrated that grooming can be interchanged for support in agonistic encounters (Seyfarth & Cheney, 1984), mating opportunities (Barrett & Henzi, 2001; Gumert, 2007b), tolerance (Gumert & Ho, 2008), food (Watts, 2002), and infant handling (Gumert, 2007a; Henzi & Barrett, 2002; Tiddi et al., 2010). In tufted capuchin monkeys (*Cebus apella nigritus*), Tiddi et al. (2010) proposed that grooming is interchanged for infant handling among females, with grooming given by potential handlers increasing the likelihood of subsequent infant handling opportunities. Similar results have also been found in wild baboons (*P. anubis*) (Frank & Silk, 2009), long-tail macaques (*Macaca fascicularis*) (Gumert, 2007a), and golden snub-nosed monkeys (Wei et al., 2013).

These previous studies suggest that both grooming and infant handling opportunities are valuable commodities in primates. Infant handling consists of an infant and adult female other than its mother. The groomer (female A) may interchange grooming to gain access to the groomee’s infant (female B, the infant’s mother). Accordingly, as a limited-competition resource, infants could be interchanged between the groomee (supplier, infant’s mother) and groomer (demander, potential handler, non-mother). However, the extent to which individuals perform grooming for infant handling or provide infant handling for grooming among females remains unclear.

We used the biological market perspective to examine the interchange relationships between grooming and infant handling in Tibetan macaques (*M. thibetana*). Tibetan macaques live in multi-male-multi-female social groups (mean group size=33.8±6.79), with female philopatry and male dispersion (Li, 1999). Tibetan macaques devote approximately 20% of their daily activity budget to grooming and thus it can be considered as a behavioral commodity in their social groups (Xia et al., 2012, 2013). Similar to other macaque species, group members persistently attempt to interact with infants in multiple ways (Li, 1999).

We hypothesized the existence of bidirectional and complex interchange patterns between grooming and infant handling in Tibetan macaques. We tested the following predictions: if an infant is a limited and valuable commodity, (1) females with infants will be more attractive than females without infants, and females will receive more but provide less grooming after giving birth than before parturition; (2) with an increase in the number of infants, females with infants will receive less grooming from females without infants; (3) females with infants will receive more grooming if they first allow their infant to be handled; if grooming is an effective way to gain access to an infant, (4) females will obtain more infant handling opportunities after grooming the infant’s mother than they have under baseline social conditions.

## MATERIALS AND METHODS

### Study site and subjects

This study was conducted at Mt. Huangshan National Reserve in Anhui Province, China. The reserve is a World Culture and Nature Heritage site and well-known tourist destination, as well as research site for the study of Tibetan macaques.

The study site can be found within the reserve in an area known as the “Valley of the Monkeys”. There are two groups at the site: Yulingkeng A1 (YA1, target group in this study) and Yulingkeng A2 (YA2). Matrilineal kinship is known from historic and demographic data collected daily since 1986 (Li, 1999). The target group has been thoroughly habituated to close observation (i.e., from <1 m) and all individuals can be recognized using physical features (i.e., scars, hair color patterns, or facial/body contours) without disturbance or capture. During the study period, the group consisted of eleven adult males, thirteen adult females, five subadults/juveniles, thirteen yearlings, and eleven infants ≤6-months-old (Table 1). We selected thirteen adult females and two subadult females for this study.

**Table 1:** Birth records of infants in the YA1 group during the study period

No.	Name	Date of birth	Date of death	Sex	Mother
1	TQY	2016–03–04	N/A	F	TXX
2	TFH	2016–03–27	N/A	F	THY
3	YXC	2016–04–16	2016–10–04	M	YCY
4	THL	2016–05–18	N/A	F	TRY
5	YXY	2016–05–29	2016–10–21	M	YH
6	TQYE	2016–06–05	2016–10–27	F	TXH
7	TXT	2016–09–18	2016–12–20	M	TH
8	HXY	2017–02–15	N/A	F	HH
9	THR	2017–02–16	2017–04–15	M	TR
10	YXM	2017–03–11	N/A	M	YCLA
11	YXD	2017–04–17	N/A	F	YCY

N/A: Not available. M: Male; F: Female.

### Data collection

This study was conducted from July 2016 to January 2017 and from March to May 2017. All behavioral data were collected during an intensive study period over 201 days (average=25.1day/month, range=24–27). The social group was followed from dawn to dusk, and behavioral observations began at approximately 0700–0800 h and ended at 1700–1800 h each day (depending on the time of year). The observer maintained an observation distance of 5–10 m from the monkeys.

Focal animal sampling and continuous recording (using a digital voice recorder) were used to score the daily activity of the focal individual (Altmann, 1974), with the recorded data used as the baseline. Focal sample duration was set at 15 min (Li et al., 2007). We followed the sampling rules of Xia et al. (2012, 2013) to avoid the influence of humans and double-counting social interactions.

To investigate whether non-mothers were more likely to handle infants after grooming their mothers, we collected post-grooming (PG) samples. The PG samples consisted of 15-min focal observations of a non-mother who had just groomed a mother. To investigate whether infant handling by non-mothers increased subsequent grooming investment given to mothers, we collected post-infant handling (PH) samples. The PH samples consisted of 15-min focal observations of a non-mother who had just handled an infant of a mother. On the next day, at the same time, we conducted 15-min matched control (MC) focal observations matched with a PG or PH sample, with no grooming or infant handling preceding the focal observation (De Waal & Yoshihara, 1983). We collected MC samples only if the two participants were in proximity (within 5 m). If the two participants were not in proximity during the MC observations and/or were involved in handling or grooming interactions within the 5 min preceding a planned MC or in the first 5 min of an ongoing MC, we postponed the MC until all conditions were meet. If a MC observation could not be conducted within one week of the PG or PH, the PG or PH was discarded.

We used *ad libitum* sampling to collect data on duration of all grooming bouts between mothers and non-mothers that involved infant handling. We categorized an interaction as a grooming-infant handling interchange if a non-mother groomed a mother and handled her infant. This could occur in any order and any number of times. The interaction finished when the two individuals moved >5 m away from each other. We measured the total duration of grooming until the two females departed each other. We recorded the dyadic social relationships immediately when an interaction ended. According to Xia et al. (2012; 2013), we defined grooming as any act in which a macaque (groomer) used its hand or mouth to touch, clean, or manipulate the fur of another individual (groomee). Infant handling behaviors included inspect, teeth-chatter, hold, groom, and bridge (Table 2).

**Table 2:** Definitions of infant handling behaviors

Behavior	Definition
Inspect	Handler brought its face within 15 cm of an infant and peered at it or smelt the genital area.
Teeth-chatter	Handler was in proximity to an infant and made clicking sounds with their teeth toward the infant.
Hold	Handler grabbed an infant using one or both hands.
Groom	Handler manipulated an infant’s hair with its hand and/or mouth (except for momentary touching), sometimes removing and eating small items found in the infant’s fur.
Bridge	Handler in proximity to the mother and glancing at the infant, with the infant carried by either the mother or handler. The pair held the infant between them and simultaneously licked the infant’s genitals or body while teeth-chattering vigorously.

Infant handling behavior definitions were modified from Manson (1999) and Li (1999).

We used *ad libitum* sampling to record aggressive and submissive behaviors to determine dominance relationships. Aggressive interactions were defined as an individual threatening, chasing, slapping, grabbing, or biting another individual (Berman et al., 2007). Submissive behaviors were scored when an individual showed fearful interactions, such as fear grin, cower, mock leave, avoid, flee, or scream (Berman et al., 2004). All records of agonism were tallied for each focal female and divided into aggression given and aggression received.

### Data analysis

We reported baseline data as mean±*SE* grooming duration (min/h) and infant handling frequency (times/h). We used a one sample Kolmogorov-Smirnov test to examine whether the data conformed to normal distribution (*P*>0.05).

We used the baseline data and calculated the duration of grooming received by mothers from non-mothers and given to non-mothers by mothers before parturition and six months after giving birth. Data were compared using paired *t*-tests to analyze whether mothers were groomed for longer periods after giving birth than before parturition, and whether mothers groomed non-mothers less after giving birth than before parturition.

We collected data on 11 of the 15 females investigated for PG and PH. No data were collected for three adult females (TT, YZ, YM), who were older and less socially active in regard to infant handling and did not give birth or become pregnant during the study. One subadult female (THX) was not observed to engage in a grooming-infant handling interchange. We used paired *t*-test to compare the duration of grooming between focal samples and PH samples, and between MC samples and PH samples to determine if non-mothers handling infants promoted the non-mothers to groom mothers. We used paired *t*-test to compare the frequency of infant handling between focal samples and PG samples, and between MC samples and PG samples to determine if non-mothers grooming mothers promoted non-mother infant handling.

We used the *ad libitum* data collected on grooming-infant handling interchange to investigate whether the number of infants per female was negatively correlated with duration of non-mothers grooming mothers during the interaction. We used linear regression to determine relationships between grooming and the number of infants per female.

We analyzed grooming-infant handling interchange data to elucidate the effect of infant age on the duration of non-mothers grooming mothers during the interaction. We used linear regression to determine the relationship between infant age and duration of non-mothers grooming mothers. We assessed individual dominance rank by calculating David’s Score (DS). We also calculated linearity for the obtained dominance hierarchy (De Vries, 1995; Gammell et al., 2003) Rank distance is the number of individuals ranking between the focal animal and a given partner, plus 1 (Castles et al., 1999). In our study, the rank distance was the number of individuals ranking between the mother and non-mother, using the mother’s rank as the standard. We determined the sequence of social ranks based on the DS values, according to Gammell et al. (2003). YH was the highest ranked, followed by YXX, YCY, YM, TXH, TH, TXX, YCLA, HH, TR, TRY, THX, TT, YZ, and THY.

We analyzed grooming-infant handling interchange data and used linear regression analysis to test the relationship between rank distance and duration of non-mothers grooming mothers. To investigate whether kinship between non-mother and mother affected the duration of non-mothers grooming mothers when infant handling, we used paired *t*-tests to compare the duration of non-mothers grooming mothers between kin and non-kin.

To account for potential bias caused by pseudoreplication, we used factorial ANOVA to test for variation across individuals and number of infants, infant age, and rank distance on the duration of non-mothers grooming mothers. We found a significant effect of number of infants, infant age, and rank distance on grooming duration, but no significant effect on individuals or interaction effects. We found no evidence that individual variation existed and therefore no support that individual idiosyncrasies solely accounted for the results. Thus, pseudoreplication did not seriously affect the results.

## RESULTS

During the study period, we collected 90 valid PG-MC samples, 86 valid PH-MC samples, and 148 grooming-infant handling interchanges.

### Grooming variation among females

**Figure 1:**
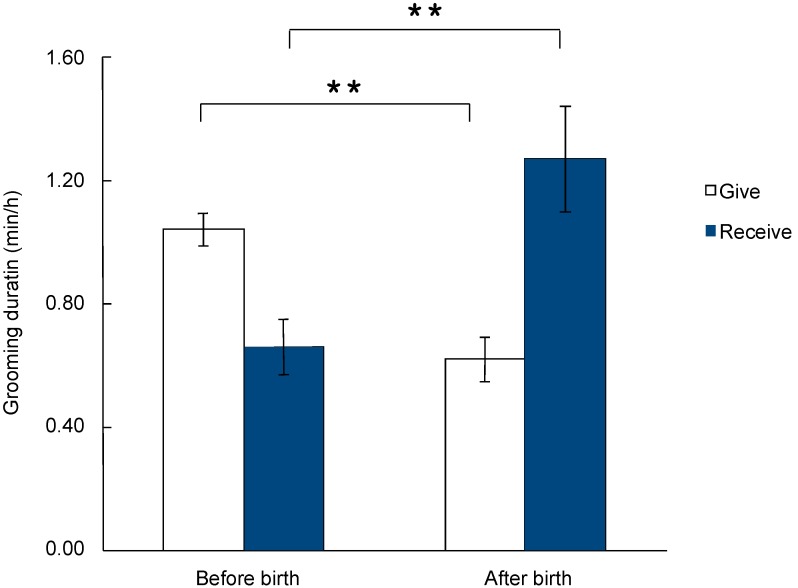
Duration of grooming females gave and received before birth and after birth (mean±*SE*)

The mean duration of mothers grooming non-mothers before birth (1.04±0.05 min/h) (mean±*SE*) was significantly longer than after birth (0.62±0.07 min/h) (paired *t*-test: *t*=6.233, *df*=5, *P*=0.002), whereas the mean duration of non-mothers grooming mothers after birth (1.27±0.17 min/h) was significantly longer than that before birth (0.66±0.09 min/h) (paired *t*-test: *t*=–4.218, *df*=5, *P*=0.008) (Figure 1).

**: *P*<0.01.

### Grooming variation between PH and baseline

**Figure 2:**
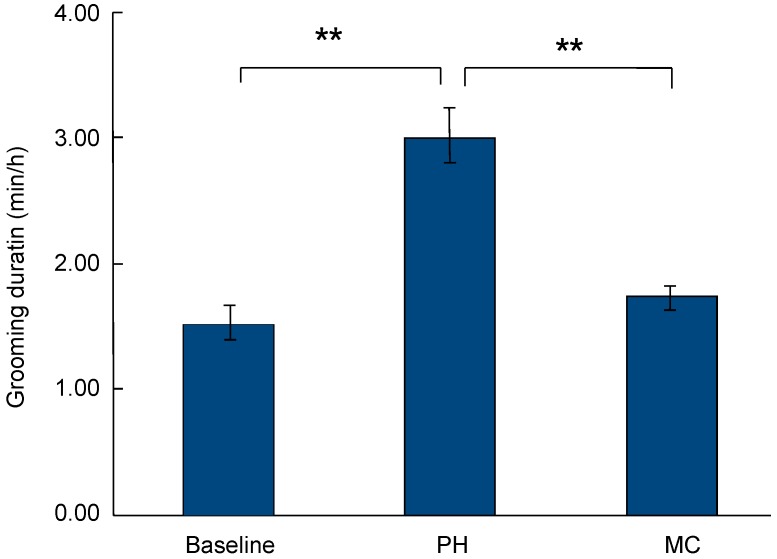
Duration of non-mothers grooming mothers (mean±*SE*)

For focal sample observations, the mean duration of non-mothers grooming mothers was 1.52±0.13 min/h (mean±*SE*). After non-mothers handled infants of mothers, the mean duration of non-mothers grooming mothers was 3.02±0.22 min/h (mean±*SE*). The duration of grooming was significantly higher in PH samples than in focal samples (paired *t*-test: *t*=7.679, *df*=10, *P*<0.001). In MC sample observations, the mean duration of non-mothers grooming mothers was 1.74±0.08 min/h (mean±*SE*). The duration of grooming was significantly higher in PH samples than in MC samples (paired *t*-test: *t*=6.475, *df*=10, *P*<0.001) (Figure 2).

Baseline represents the mean from focal sample data. PH represents the mean from post-infant handling sample data. MC represents the mean from MC sample data where mothers and non-mothers were in proximity. **: *P*<0.01.

### Grooming variation with number of infants

**Figure 3:**
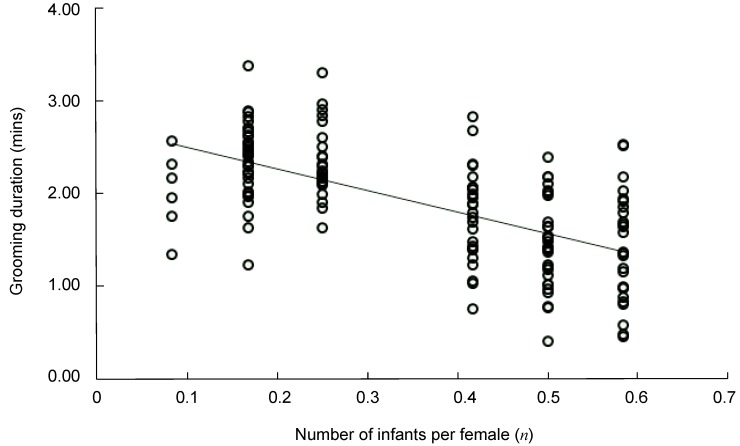
Duration of non-mothers grooming mothers was negatively correlated with the number of infants per female (mean±*SE*)

The number of infants per female was negatively correlated with the duration of non-mothers grooming mothers during grooming-infant handling interchange (linear regression: R=0.624, F=93.271, *P*<0.001, R^2^=0.390, *df*=147) (Figure 3).

Linear regression: R=0.624, F=93.271, *P*<0.001, R^2^=0.390, *df*=147.

### Variation of infant handling

**Figure 4:**
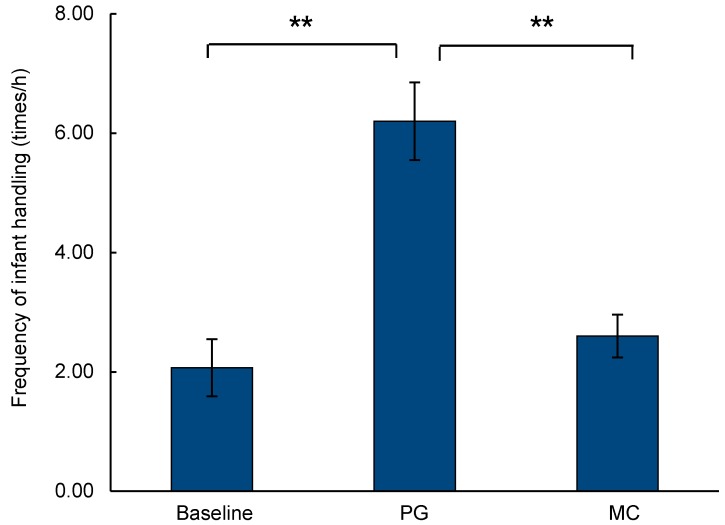
Frequency of non-mother infant handling (mean±*SE*)

In focal sample observations, the mean frequency of non-mother infant handling was 2.07±0.48 times/h (mean±*SE*). After non-mothers groomed mothers, the mean frequency of non-mother infant handling was 6.20±0.65 times/h (mean±*SE*). The mean frequency of infant handling was significantly higher in PG samples than in focal samples (paired *t*-test: *t*=7.642, *df*=10, *P*<0.001). In MC sample observations, the mean frequency of infant handling was 2.60±0.36 times/h (mean±*SE*). The mean frequency of infant handling was significantly higher in PG samples than in MC samples (paired *t*-test: *t*=5.858, *df*=10, *P*<0.001) (Figure 4).

Baseline represents the mean from focal sample data, PG represents the mean from post-grooming sample data, MC represents the mean from MC sample data where mothers and non-mothers were in proximity. **: *P*<0.01.

### Effect of infant age

**Figure 5:**
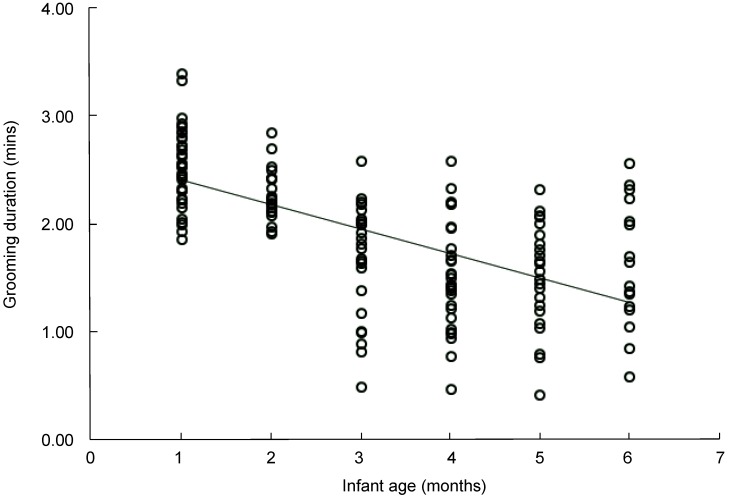
Duration of non-mothers grooming mothers was negatively correlated with infant age (mean±*SE*)

Infant age was negatively correlated with the duration of non-mothers grooming mothers during grooming-infant handling interchanges (linear regression: R=0.634, F=98.128, *P*<0.001, R^2^=0.402, *df*=147) (Figure 5). The duration of non-mothers grooming mothers decreased significantly with infant age.

Linear regression: R=0.634, F=98.128, *P*<0.001, R^2^=0.402, *df*=147.

### Effect of rank and kin

**Figure 6:**
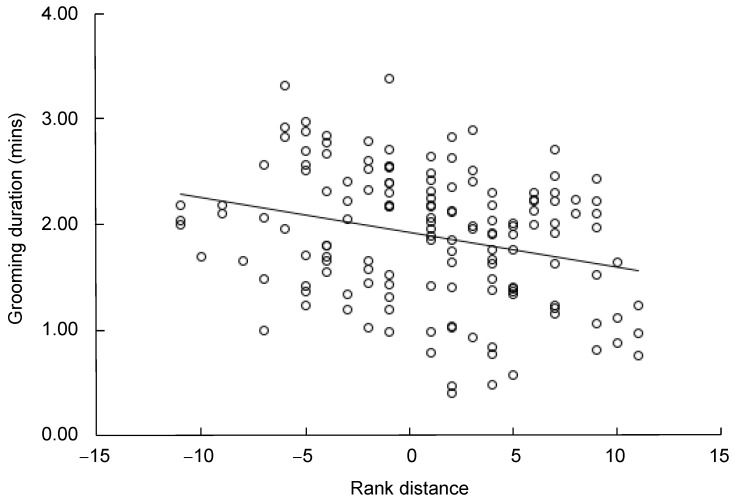
Duration of non-mothers grooming mothers was negatively correlated with rank distance between mothers and non-mothers (mean±*SE*)

The rank distance between mothers and non-mothers was negatively correlated with the duration of non-mothers grooming mothers (linear regression: R=0.277, F=12.088, *P*=0.001, R^2^=0.076, *df*=147) (Figure 6).

Linear regression: R=0.277, F=12.088, *P*=0.001, R^2^=0.076, *df*=147.

There was no significant difference in the duration of non-mothers grooming mothers when infant handling between kin and non-kin (paired *t*-test: *t*=0.758, *df*=4, *P*=0.491).

## DISCUSSION

Infants are considered as commodities between a groomee (supplier, infant’s mother) and groomer (demander, potential handler, non-mother). However, the extent to which individuals groom in exchange for infant handling or offer infant handling in exchange for grooming among females remains unclear. In this study, we found that mothers received more grooming from non-mothers after birth than before birth, and that mothers groomed non-mothers less after birth than before birth. These results supported the prediction that females with infants are more attractive than females without infants and that females will receive more but provide less grooming after birth than before parturition. These findings are similar to that reported for golden snub-nosed monkeys (*R. roxellana*) (Wei et al., 2013). Furthermore, after non-mothers handled infants, the duration of non-mothers grooming mothers was significantly higher than that observed under baseline conditions, supporting the prediction that females with infants will receive more grooming if they first allow their infant to be handled. These results are similar to that reported for wild baboons (*P. anubis*) (Frank & Silk, 2009). We also demonstrated positive evidence for the prediction that females will gain higher infant handling opportunities after grooming an infant’s mother compared with baseline interactions. Similar results have also been found in studies on golden snub-nosed monkeys (*R. roxellana*) and long-tailed macaques (*M. fascicularis*) (Gumert, 2007a; Yu et al., 2013). Thus, we concluded that infants are a limited and valuable commodity and can be used by mothers in exchange for grooming. Furthermore, grooming is an effective way for non-mothers to access infants. Our results showed bidirectional and complex interchange patterns between grooming and infant handling in Tibetan macaques, and that the adaptive investment of females will change based on their social characteristics and group dynamics.

A single behavioral exchange may not explain all behavioral relationships among individuals. In the case of reciprocity, pairs of actors are expected to form long-term and predictable social relationships so that, on average, the overall costs and benefits to each are relatively equal over time (Seyfarth, 1977; Xia et al., 2012). In contrast, under conditions in which individual actors have the opportunity to interact with a diverse set of social partners who vary in the quality of goods or services each can provide at any given moment in time, individuals are expected to seek out partners who provide the greatest benefit at the lowest cost (Noë et al., 1991; Wilkinson, 1984). These interactions can involve an exchange of the same goods or services (i.e., grooming in exchange for grooming) or the interchange of different goods or services. In Tibetan macaques, grooming can be exchanged for grooming (Xia et al., 2012, 2013) and interchanged for tolerance (Xia et al., 2012). In our study, grooming was interchanged for infant handling. However, we suggest that a single exchange may not fully explain the behavioral relationship between individuals. The behavioral exchange model is complex, reflecting the evolution of individual adaptability and promoting relationship maintenance and group stability in primate societies.

Market forces affect the interchange between grooming and infant handling. Infant age is a market force that can influence grooming payment. In our study, Tibetan macaque females groom other females for a longer period when they have a younger infant, with similar results also reported in studies on long-tailed macaques (*M. fascicularis*), tufted capuchin monkeys (*Cebus apella nigritus*), sooty mangabeys (*Cercocebus atys*), and vervet monkeys (*Chlorocebus aethiops*) (Fruteau et al., 2011; Gumert, 2007a; Tiddi et al., 2010). These results reflect that newborn infants are generally more attractive than older infants (Nicolson, 1987), with infants get older, their attractiveness declined. Market theory predicts that supply and demand will influence behavior exchange among group members by altering the value of the commodities traded (Noë et al., 2001). Under a biological market perspective, potential handlers attracted to infants represent the demand and infants represent the supply (Gumert, 2007a; Henzi & Barrett, 2002). In Tibetan macaques, with the increase in the number of infants, the duration of non-mothers grooming mothers for infant handling decreased significantly. Similar results have been reported in studies on chacma baboons (*P. cynocephalus ursinus*) and long-tailed macaques (*M. fascicularis*) (Gumert, 2007a; Henzi & Barrett, 2002).

Dominance hierarchy is a factor that has considerable influence on social interchange in non-human primates. Dominance relationships may make social commodities cheaper for higher-ranked individuals and more expensive for lower-ranked individuals (Gumert, 2007a; Henzi & Barrett, 2002; Tiddi et al., 2010; Wei et al., 2012). In Tibetan macaques, rank can affect the patterns of grooming reciprocity among females; within the context of a biological market, higher ranking females have a wider set of social options than middle and lower ranking females (Xia et al., 2012). Our study indicated that dominance also altered the interchange between grooming and infant handling in Tibetan macaques, by reducing the grooming investment that higher ranking non-mothers gave to mothers when infant handling and increasing the grooming investment that lower ranking non-mothers gave to mothers. These results indicate that, in an infant market, lower-ranked individuals will pay more for interacting with higher-ranked partners, and rank might corrupt a social market and skew social exchange to benefit higher-ranked individuals.

In Tibetan macaques, we found the interchange between grooming and infant handling to be bidirectional and to change with the age and number of infants. The interchange relationship between grooming and infant handling is an important part of social relationship maintenance and group stability in primate societies. It not only helps us to understand the characteristics of behavioral adaptation in adult individuals, but also highlights the social functions of infants.
